# Arterial Stiffness in a Cohort of Young People Living With Perinatal HIV and HIV Negative Young People in England

**DOI:** 10.3389/fcvm.2022.821568

**Published:** 2022-03-01

**Authors:** J. Mellin, M. Le Prevost, J. Kenny, K. Sturgeon, L. C. Thompson, C. Foster, H. H. Kessler, Nandu Goswami, N. Klein, A. Judd, H. Castro

**Affiliations:** ^1^Gravitational Physiology and Medicine Research Unit, Division of Physiology, Otto Loewi Research Center, Medical University of Graz, Graz, Austria; ^2^Medical Research Council Clinical Trials Unit at University College London, London, United Kingdom; ^3^Guy's and St. Thomas' National Heath Service Foundation Trust, Evelina London Children's Hospital, St. Thomas' Hospital, London, United Kingdom; ^4^Imperial College Healthcare National Heath Service Trust, St. Mary's Hospital, London, United Kingdom; ^5^Diagnostic and Research Institute of Hygiene, Microbiology, and Environmental Medicine, Medical University of Graz, Graz, Austria; ^6^Mohammed Bin Rashid University of Medicine and Health Sciences, Dubai, United Arab Emirates; ^7^Department of Infection, Immunity and Inflammation, University College London Great Ormond Street Institute of Child Health, London, United Kingdom

**Keywords:** HIV, perinatal, pulse wave velocity, arterial stiffness, young people, England, vascular function, cardiovascular disease

## Abstract

**Background:**

Antiretroviral therapy (ART) has increased life expectancy and consequently the risk of cardiovascular disease (CVD) in adults living with HIV. We investigated the levels and predictors of arterial stiffness in young people (YP) living with perinatal HIV (PHIV) and HIV negative YP in the Adolescents and Adults Living with Perinatal HIV (AALPHI) study.

**Methods:**

AALPHI was a prospective study evaluating the impact of HIV infection and exposure to ART on YP living with PHIV (aged 13–21 years) who had known their HIV status for at least 6 months, and HIV negative YP (aged 13–23 years) who either had a sibling, friend or parent living with HIV. Participants were enrolled from HIV clinics and community services in England. Two hundred and thirteen PHIV and 65 HIV negative YP (42% siblings of PHIV) had pulse wave velocity (PWV) measurements taken (Vicorder software) from the supra-sternal notch to the middle of the thigh cuff, at their second interview in the study between 2015 and 2017. Average PWV was calculated from the three closest readings (≥3 and ≤ 12 m/s) within 0.6 m/s of each other. Linear regression examined predictors of higher (worse) PWV, including age, sex, HIV status and height as *a priori*, ethnicity, born outside UK/Ireland, alcohol/nicotine/drug use, weight, waist-to-hip-ratio, mean arterial pressure (MAP), caffeine 2 h before PWV and nicotine on day of PWV. A separate PHIV model included CD4, viral load, years taking ART and ART regimen.

**Findings:**

One hundred and twenty eight (60%) PHIV and 45 (69%) HIV negative YP were female (*p* = 0.18), with median (IQR) age 18 (16, 20) and 18 (16, 21) years (*p* = 0.48) respectively. Most PHIV were taking a combination of three ART drugs from two classes. There was a trend toward higher (worse) mean PWV in the PHIV group than the HIV negative group [unvariable analysis 6.15 (SD 0.83) m/s vs. 5.93 (0.70) m/s, respectively, unadjusted *p* = 0.058], which was statistically significant in the multivariable analysis [adjusted *p* (ap) = 0.020]. In multivariable analysis being male (ap = 0.002), older age (ap < 0.001), higher MAP (ap < 0.001) and nicotine use on day of measurement (ap = 0.001) were also predictors of higher PWV. The predictors were the same in the PHIV model.

**Interpretation:**

By late adolescence PHIV had worse PWV in comparison to HIV negative peers, and traditional risk factors for CVD (higher arterial pressure, being male and older age) were associated with higher PWV values. Regular detailed monitoring of cardiovascular risk factors should become standard of care for every young person with PHIV worldwide.

## Introduction

Combination antiretroviral therapy has turned human immunodeficiency virus (HIV) into a manageable chronic condition for the 38 million people living with HIV today, of whom about 1.7 million (5%) are young people aged 10–19 years (1, 2).

However, an increased life expectancy has consequently increased the incidence of non-communicable conditions. Studies in adults living with HIV have shown an increased risk of cardiovascular disease (CVD) (3). The underlying mechanisms are based on an interplay of an increased prevalence of traditional or conventional cardiovascular risk factors, adverse side effects of antiretroviral therapy (ART), “emerging” risk factors based on chronic inflammation and infection, as well as a changed immunology due to the HIV infection itself, that lead to a proinflammatory state promoting endothelial dysfunction and atherosclerotic changes to the vasculature (4). While children and adolescents might not have signs of symptomatic atherosclerosis yet, precursors of atherosclerosis such as endothelial dysfunction as well as changes of the vascular wall and arterial stiffness have been shown as early as the first decade of life (5). Therefore, while AIDS related mortality has decreased in children since the introduction of ART, focus has shifted toward non-AIDS morbidity due to possible accelerated progression of atherosclerosis and therefore premature aging (3).

Vascular function can be measured using various techniques; pulse wave velocity (PWV) is a dynamic parameter of arterial stiffness (reciprocal of arterial distensibility) that describes the rate at which the pulse wave travels over a given distance of the arterial tree. The speed is determined by the elastic and geometric properties of the arterial wall and is also dependent on rheological characteristics of the blood, ventricular function and blood pressure (6). Therefore, increased vascular damage and arterial stiffness lead to an increased (worse) pulse wave velocity which has been shown to predict worse cardiovascular outcomes in adults (7).

The actual risk of cardiovascular outcomes in young people living with perinatal HIV (PHIV), who (with ART) are surviving well into their thirties and forties, is still unclear. Therefore, the early and regular assessment of cardiovascular risk, due to HIV, ART and the increasing prevalence of traditional risk factors such as hypertension and obesity in adolescents, becomes increasingly important in the care and management of these young people (8).

This paper explores the levels and predictors of arterial stiffness in young people living with PHIV and HIV negative young people, in the largest study measuring arterial stiffness in PHIV to date. The analyses are exploratory, given the unclear evidence base in this group.

## Methods

### Study Population and Design

The Adolescents and Adults Living with Perinatal HIV (AALPHI) cohort was a prospective study evaluating the impact of HIV infection and exposure to ART on young people living with PHIV in England and comparing outcomes with HIV negative young people, across several research areas, including cognitive function, anxiety and depression, sexual and reproductive health, self-harm and adherence. Participants in both groups were enrolled from HIV clinics and community organisations (providing services for PHIV young people and HIV negative young people affected by HIV) in England and undertook first interviews from 2013 and 2015, and second interviews between 2015 and 2017. Detailed methods have been described previously (9). Full ethical approval was obtained from the Leicester Research Ethics Committee. All participants living with PHIV had known their HIV status for at least 6 months, were aged 13–21 years, and were all included in the national UK and Ireland Collaborative HIV Pediatric Study (CHIPS) (10). HIV negative young people were aged 13–23 years, and either had a sibling, friend or parent living with HIV. All participants had lived in the UK for 6 months or longer, and could speak and understand English. This analysis examines vascular function measured in PHIV young people compared to HIV negative young people, measured at their second interview. The variables used in the analysis are described below and were measured at interview two, unless stated otherwise.

### Anthropometric Measurements

Weight and height as well as hip and waist circumference were measured. Weight, height, BMI and waist to hip ratio z-scores were calculated with reference to HIV negative young people, adjusted for age and sex (11–13). The Microlife blood pressure monitor BP 3AG1 was used for all blood pressure and heart rate readings. Average blood pressure (BP) as well as average heart rate were calculated from two measurements at the time of the pulse wave velocity assessment, and used to calculate mean arterial pressure (MAP). Systolic BP (SBP) and diastolic BP (DBP) z-scores were calculated with reference to HIV negative young people, adjusted for age and sex (14).

### Risk Factor Assessment

Traditional cardiovascular risk factors and contributing factors such as sex, positive family (biological father, mother, brother or sister) history (reported at interview one) of CVD (stroke/high blood pressure/heart disease/heart attack) and metabolic changes (diabetes type I/diabetes type II/high levels of fat in blood), previous medical conditions in the young person (apart from HIV, reported at interview one), face recognition (normal/mild/moderate/severe facial lipoatrophy (visual scale) reported at interview one), frequency of exercise (five or more times a week/one to four times a week/one to three times a month/less than once a month/never in the last year), smoking, alcohol and recreational drug consumption were self-reported on case report forms for both groups. Additionally, the obtained anthropometric measurements were used for the classification obesity (13). Hazardous drinking was defined by the WHO Alcohol Use Disorders Identification Test (15).

### Pulse Wave Velocity

The pulse wave velocity (PWV) measurements were all undertaken with the Vicorder device and software, at the second interview. The oscillometric pressure cuffs were placed at the carotid as well as the right thigh (as high as possible); the most commonly used measuring sites of PWV. The measurements were taken after a 10-min resting time in supine position with an upper body elevation of 30°. To determine the distance between the carotid and the femoral arteries the length was taken from the suprasternal notch to the middle of the right thigh cuff. After the inflation of the cuffs, the pressure waveforms over the right carotid as well as the right thigh were simultaneously recorded. The Vicorder software subsequently provided the pulse wave velocity measurement in meters/second. Average PWV was calculated as the average of the closest 3 readings (of altogether six readings) within 0.6 m/s of each other (≥3 and ≤ 12 m/s).

### HIV Infection Markers and Lipid Panel

As the PHIV participants in AALPHI were also in CHIPS (for which detailed clinical data are annually collected from patient notes), HIV infection markers (RNA viral load, CD4 count), ART exposure and lipid panel markers were obtained where possible, as well as Centers for Disease Control and Prevention (CDC) worst stage and subtype (16). Lipid levels were categorized as acceptable, borderline high or high, according to age-appropriate cut-offs (17). Lipid levels were not available for HIV negative participants.

### Statistical Analyses

Data were analyzed using STATA version 16.1 (Stata Corp, College Station, Texas, USA). Characteristics of participants were compared between the two groups using the Chi squared test for proportions and Wilcoxon rank sum for medians, and a *p*-value < 0.05 was considered statistically significant. Results are presented for non-missing values; missing values were <10% of study participants unless specified. The effect of potential predictors of higher (worse) PWV was explored using linear regression. Age, sex, HIV status and height were considered *a priori* factors associated with PWV. Other variables considered were ethnicity, country of birth (within vs. outside the UK/Ireland), death of one or both parents, positive family history of CVD and metabolic changes, ever smoked, ever consumed alcohol, ever taken recreational drugs, mental health (Hospital Anxiety and Depression Scale (HADS) scores in the past week, measured at interview one, ranging from 0 to 21, higher scores indicating more severe anxiety or depression (18)), how much the participant had exercised in the last year (five or more times a week/one to four times a week/one to three times a month/less than once a month/never), weight, BMI, waist to hip ratio, MAP, and on the day of the PWV measurement, hours since last meal, any caffeine 2 h before the measurement, any nicotine that day and room temperature at the time of the measurement. Variables with a *p*-value < 0.15 in the univariable models were included in the multivariable models using backwards elimination, and a *p*-value < 0.05 was considered statistically significant.

An additional analysis for only participants with PHIV also included CD4 cell count, RNA viral load <50 copies/ml, total cholesterol and triglycerides [all within 6 months before or after interview two and included as continuous variables in the analysis (apart from RNA viral load)], years taking ART, ever taken a protease inhibitor, ever taken abacavir, ART regimen at interview two [current ART status (never started ART/taking ART/not currently taking ART but previously have taken ART), number of drugs, number of ART classes], CDC stage C disease and subtype, as potential predictors. Two self-reported (*via* computer-assisted survey interviewing) ART adherence measures were also included as potential predictors; at interview two, participants were asked to report any missed doses of their HIV medication in the last 3 days (yes/no) and to rate how they felt their adherence was at the time of the interview (excellent, good, not so good or bad).

#### Role of Funding Source

The funding sources had no role in the study design, collection, analysis and interpretation of data, writing of the report or decision to submit the paper for publication.

## Results

### Study Population

A total of 278 participants (213 PHIV and 65 HIV negative) had PWV measurements at interview two. HIV negative participants either had a mother [32 (49%)] or sibling [33 (51%), of which 27 were siblings of PHIV participants] living with HIV. The median age at interview two was 18 years for both groups, there were more female than male participants in each group, the majority were black [PHIV 183 (86%), HIV negative 49 (75%), *p* = 0.046], and a higher proportion were born outside of the UK/Ireland in the PHIV group [PHIV 129 (61%), HIV negative 30 (46%), *p* = 0.040] (Table 1). The majority of participants were in education at the time of interview two. Eighty-two (41%) PHIV and 17 (28%) HIV negative participants (*p* = 0.068) had one or both parents who had died.

**Table 1:** Characteristics of PHIV and HIV negative participants.

	**PHIV (*n* = 213)**	**HIV negative (*n* = 65)**	***P-*value**
**Sociodemographics**			
Years between interview one and interview two, median (IQR)	1.8 (1.3, 2.6)	1.6 (1.2, 2.3)	0.025
Age in years at interview two, median (IQR)	18 (16, 20)	18 (16, 21)	0.482
Age at interview two, no. (%)			0.096
≤15 years	29 (13.6%)	16 (24.6%)	
16–18 years	94 (44.1%)	23 (35.4%)	
≥19 years	90 (42.3%)	26 (40.0%)	
Sex, no. (%)			0.184
Female	128 (60.1%)	45 (69.2%)	
Male	85 (39.9%)	20 (30.8%)	
Ethnicity (black), no. (%)	183 (85.9%)	49 (75.4%)	0.046
Born outside UK/Ireland, no. (%)	129 (60.6%)	30 (46.2%)	0.040
Death of one/both parents, no. (%)	82 (40.8%)	17 (27.9%)	0.068
Occupation at interview two, no (%)			0.778
Education, no. (%)	163 (76.9%)	51 (78.5%)	
Employment, no. (%)	36 (17.0%)	9 (13.9%)	
Not in education/employment, no. (%)	13 (6.1%)	5 (7.7%)	
**Body composition at interview two**			
Height in meters, median (IQR)	1.65 (1.57, 1.72)	1.66 (1.60, 1.74)	0.066
Height z-score, median (IQR)	−0.51 (−1.37, 0.26)	0.29 (-0.75, 0.77)	<0.001
Weight in kilograms, median (IQR)	63.0 (54.0, 75.0)	67.4 (60.0, 80.7)	0.018
Weight z-score, median (IQR)	0.41 (−0.74, 1.24)	1.03 (0.17, 1.74)	0.001
BMI, median (IQR)	23.2 (20.5, 26.8)	24.7 (22.0, 28.6)	0.050
BMI z-score, median (IQR)	0.61 (−0.28, 1.49)	1.05 (0.17, 1.93)	0.028
Waist to hip ratio, median (IQR)	0.87 (0.81, 0.94)	0.85 (0.77, 0.93)	0.249
Waist to hip ratio z-score, median (IQR)	1.37 (0.36, 2.30)	1.15 (0.19, 2.09)	0.487
**Obesity Classification, no. (%)**			0.233
Underweight, no. (%)	3 (1.5%)	0	
Healthy BMI, no. (%)	128 (61.8%)	27 (49.1%)	
Overweight, no. (%)	51 (24.6%)	18 (32.7%)	
Obese, no. (%)	25 (12.1%)	10 (18.2%)	
Blood pressure^*^and heart rate^*^at interview two			
SBP in mmHg, median (IQR)	114 (107, 121)	114 (110, 123)	0.270
SBP z-score, median (IQR)	−0.06 (−0.61, 0.76)	0.04 (−0.33, 0.72)	0.275
DBP in mmHg, median (IQR)	68 (63, 74)	71 (66, 77)	0.043
DBP z-score, median (IQR)	0.19 (−0.30, 0.72)	0.37 (−0.14, 0.87)	0.104
Average MAP, median (IQR)	83 (78, 89)	85 (81, 91)	0.050
Average heart rate, median (IQR)	71 (63, 77)	72 (63, 77)	0.974
**Other risk factors**			
Positive family history of CVD/metabolic changes^a^, no. (%)	78 (43.1%)	29 (46.8%)	0.614
Ever smoked, no. (%)	79 (37.4%)	25 (39.1%)	0.815
Ever consumed alcohol, no. (%)	132 (62.3%)	43 (66.2%)	0.569
Hazardous drinking in the last year, no. (%)	34 (16.1%)	5 (7.9%)	0.103
Ever used recreational drugs, no. (%)	55 (27.9%)	22 (37.3%)	0.169
Exercised in the last year, no. (%)			0.344
1–3 times a month or less	64 (30.1%)	14 (21.5%)	
1–4 times a week	114 (53.5%)	41 (63.1%)	
≥5 times a week	35 (16.4%)	10 (15.4%)	
HADS anxiety score, median (IQR)	6.0 (3.0, 9.0)	6.0 (3.0, 8.0)	0.417
HADS anxiety grouped score, no. %			0.344
Normal, no. (%)	118 (60.8%)	42 (68.9%)	
Mild, no. (%)	41 (21.1%)	10 (16.4%)	
Moderate, no. (%)	28 (14.4%)	5 (8.2%)	
Severe, no. (%)	7 (3.6%)	4 (6.6%)	
HADS depression score, median (IQR)	3.0 (1.0, 6.0)	2.0 (1.0, 5.0)	0.132
HADS depression grouped score, no. (%)			0.119
Normal, no. (%)	160 (82.5%)	52 (85.3%)	
Mild, no. (%)	22 (11.3%)	9 (14.8%)	
Moderate, no. (%)	12 (6.2%)	0	
Severe, no. (%)	0	0	
**Medical history**			
Prematurity^b^, no. (%)	28 (21.5%)	10 (22.2%)	0.924
Diabetes type I, no. (%)	0	0	…
Diabetes type II, no. (%)	0	0	…
High blood pressure, no. (%)	2 (1.0%)	2 (3.2%)	0.208
High levels of fat in blood, no. (%)	8 (4.0%)	2 (3.2%)	0.791
Heart disease, no. (%)	1 (0.5%)	0	0.579
Rheumatoid arthritis, no. (%)	2 (1.0%)	1 (1.6%)	0.684
Kidney problems, no. (%)	7 (3.4%)	0	0.138
Liver problems, no. (%)	7 (3.4%)	0	0.135
Face recognition (mild/moderate/severe lipoatrophy), no. (%)	6 (2.9%)	0	0.173
**Measurements on the day of the PWV assessment**
Hours since last meal, median (IQR)	4.5 (2.5, 8.4)	4.1 (3.0, 12.4)	0.812
Room temperature in degrees Celsius, median (IQR)	22.1 (20.8, 23.3)	21.6 (20.0, 23.0)	0.129
Had caffeine in last 2 h, no. (%)	25 (11.7%)	12 (18.8%)	0.148
Had nicotine today, no. (%)	17 (8.0%)	5 (7.8%)	0.965
PWV measurement in m/s., mean (SD)	6.15 (0.83)	5.93 (0.70)	0.058

*PHIV, perinatal HIV; IQR, interquartile range; CVD, cardiovascular disease; HADS, Hospital Anxiety and Depression Scale; BMI, body mass index; SBP, systolic blood pressure; DBP diastolic blood pressure; MAP, mean arterial pressure; PWV, pulse wave velocity*.

a *Unknown for 32 PHIV and 3 HIV negative*.

b *Unknown for 83 PHIV and 20 HIV negative*.

* *Average of two measurements*.

### Blood Pressure and Anthropometric Measurements

HIV negative participants were taller and heavier than PHIV participants (Table 1). Overall ~60% of all participants had a healthy BMI [PHIV 128 (62%), HIV negative 27 (49%)] and similar proportions in each group were overweight [PHIV 51 (25%), HIV negative 18 (33%)] and obese [PHIV 25 (12%), HIV negative 10 (18%)]. Waist-to-hip ratio was similar across groups, as was SBP. DBP and consequently MAP were slightly higher for HIV negative participants, however SBP and DBP z-scores were similar for both groups.

### Other Characteristics

There were no statistically significant differences between the groups in the other characteristics examined (Table 1). Just under half the participants had a family history of CVD or metabolic changes. Almost 40% of participants had ever smoked in each group, and over 60% had ever consumed alcohol, with slightly more PHIV drinking alcohol to levels considered hazardous in the last year [PHIV 34 (16%), HIV negative 5 (8%), *p* = 0.103]. Approximately one third of participants reported to have ever used recreational drugs. Most participants had exercised 1–4 times a week in the last year. The majority of participants had normal or mild anxiety and normal depression scores, with similar numbers across both groups. Reported numbers of previous medical conditions (apart from HIV) were low in both groups.

### HIV Clinical Markers and Lipid Levels

The majority of PHIV participants were taking ART at the time of interview two; 9 (4%) participants were treatment naive and 13 (6%) were currently off treatment but had previous ART exposure (Table 2). The median age at ART initiation was 7.5 years, and the median time on ART at interview two was 9.4 years. Most PHIV participants were taking a combination of three drugs from two classes. Approximately three-quarters of PHIV participants had ever taken a protease inhibitor [156 (77%)]; similarly 150 (74%) had ever taken abacavir. Only 30 (15%) had ever taken an integrase inhibitor (15 had ever taken dolutegravir, 14 raltegravir and 2 elvitegravir). Self-reported adherence mostly ranged from excellent [69 (36%)] to good [91 (48%)], although 59 (31%) PHIV participants admitted to missing a dose in the 3 days prior to the interview.

**Table 2:** HIV clinical markers and lipids levels of PHIV participants.

	**PHIV (*N* = 213)**
**Antiretroviral therapy**	
ART status at interview two, no. (%)	
Never started ART	9 (4.2%)
Taking ART	191 (89.7%)
Currently not taking ART (but previous ART exposure)	13 (6.1%)
Age in years at initiation of ART^a^, median (IQR)	7.5 (3.0, 11.7)
Time in years on ART^a^ (excluding time off ART), median (IQR)	9.4 (5.9, 13.3)
Time in years off ART (since starting ART)^a^, median (IQR)	0.0 (0.0, 1.3)
Number of ART classes taken at interview two^b^, no. (%)	
1 class	14 (7.3%)
2 classes	168 (88.0%)
3 classes	9 (4.7%)
Number of ART drugs taken at interview two^b^, no. (%)	
1 drug	13 (6.8%)
2 drugs	21 (11.0%)
3 drugs	151 (79.1%)
4 drugs	5 (2.6%)
5 drugs	1 (0.5%)
Ever taken protease inhibitors, no. (%)	156 (76.5%)
Ever taken abacavir, no. (%)	150 (73.5%)
Ever taken integrase inhibitors, no. (%)	30 (14.7%)
**Markers of HIV infection**	
CD4 nadir (cells/mm^3^), median (IQR)	217 (128, 330)
CD4 cell count at interview two^c^, median (IQR)	560 (418, 751)
Viral load <50 copies/ml at interview two, no. (%)	129 (64.2%)
Viral load <400 copies/ml at interview two, no. (%)	151 (75.1%)
Ever CDC C, no. (%)	51 (24.0%)
HIV-1 group M subtype^>d^, no. (%)	
Subtype C, no. (%)	99 (62.7%)
Other than subtype C, no. (%)	59 (37.3%)
**Self-reported adherence at interview two**	
How would you rate your adherence?	
Excellent, no. (%)	69 (36.3%)
Good, no. (%)	91 (47.9%)
Not so good, no. (%)	19 (10.0%)
Bad, no. (%)	11 (5.8%)
Missed any doses in the past 3 days, no. (%)	59 (31.1%)
**Lipids at interview two**	
Total cholesterol in mmol/litre^e^, median (IQR)	4.20 (3.60, 4.80)
Total cholesterol categorisation^e^	
Acceptable^f^, no. (%)	80 (63.0%)
Borderline high^g^, no. (%)	30 (23.6%)
High^h^, no. (%)	17 (13.4%)
Triglycerides in mmol/litre^i^, median (IQR)	1.00 (0.72, 1.37)
Triglycerides categorisation^i^	
Acceptable^j^, no. (%)	73 (58.9%)
Borderline high^k^, no. (%)	30 (24.2%)
High^l^, no. (%)	21 (16.9%)

*PHIV, perinatal HIV; ART, antiretroviral therapy; IQR, interquartile range; CDC, Centers for Disease Control and Prevention*.

a *Not including participants who have never started ART*.

b *If a drug is low dose it is not counted*.

c *Unknown for 27 participants*.

d *Unknown for 55 participants*.

e *Unknown for 86 participants. 63/127 (49%) participants were not fasting at the time of the measurement and the fasting status is unknown for 64/127 (51%) participants*.

f *Total cholesterol <170 mg/dl for age <20 years, <190 mg/dl for age ≥20 years*.

g *Total cholesterol ≥170 mg/dl and <200 mg/dl for age <20 years, ≥190 mg/dl and <225 mg/dl for age ≥20 years*.

h *Total cholesterol ≥200 mg/dl for age <20 years, ≥225 mg/dl for age ≥20 years*.

i *Unknown for 89 participants. 62/124 (50%) participants were not fasting at the time of the measurement and the fasting status is unknown for 62/124 (50%) participants*.

j *Triglycerides <90 mg/dl for age <20 years, <115 mg/dl for age ≥20 years*.

k *Triglycerides ≥90 mg/dl and <130 mg/dl for age <20 years, ≥115 mg/dl and <150 mg/dl for age ≥20 years*.

l *Triglycerides ≥130 mg/dl for age <20 years, ≥150 mg/dl for age ≥20 years*.

Median CD4 count [within 6 months before or after the interview) was 560 cells/mm^3^ (IQR 418, 751 cells/mm^3^] and 64% had a suppressed viral load <50 copies/ml (75% had a suppressed viral load <400 copies/ml). Nearly half of the PHIV participants had subtype C virus. The median total cholesterol and triglyceride levels closest to interview two were 4.20 and 1.00 mmol/l, respectively, and ~60% were at acceptable levels for both measurements.

### Pulse Wave Velocity

On the day of the PWV measurements, the PHIV and HIV negative participants had been fasting for a median of 4.4 h, and the median room temperature was around 22°C. Similar proportions in both groups had used nicotine that day and had caffeine in the 2 h before the measurement (Table 1).

In univariable analysis, there was a trend toward higher (worse) pulse wave velocity readings in the PHIV group compared to the HIV negative group; mean (SD) average PWV was 6.15 (0.83) m/s in the PHIV group and 5.93 (0.70) m/s in the HIV negative group (unadjusted *p* = 0.058).

### Univariable and Multivariable Analysis (Tables 3, 4)

Table 3 presents univariable and multivariable predictors of higher (worse) PWV for PHIV and HIV negative participants. Both before and after adjustment for other factors, having PHIV [adjusted p (ap) = 0.020], being male (ap = 0.002), older age (ap < 0.001), higher MAP (ap < 0.001) as well as nicotine use on the day of the PWV measurement (ap = 0.001) were predictors of higher PWV. Being born outside of the UK/Ireland, ever consumed alcohol, ever smoked, ever used recreational drugs, height and consumed caffeine in the 2 h before the PWV measurements were all associated with worse PWV in univariable analyses but not in multivariable analyses.

**Table 3:** Univariable and multivariable predictors of PWV in PHIV and HIV negative young people.

	**Univariable predictors**		Multivariable predictors^**^	
	**Coefficient (95% CI)**	***P-*value**	**Coefficient (95% CI)**	***P-*value**
Constant			2.28 (0.13 to 4.43)	…
**Sociodemographics**				
PHIV (vs. HIV negative)	0.22 (−0.01 to 0.44)	0.058	0.25 (0.04 to 0.47)	0.020
Male (vs. female)	0.50 (0.31 to 0.69)	<0.001	0.37 (0.13 to 0.60)	0.002
Age, per 1 year increase	0.09 (0.05 to 0.12)	<0.001	0.07 (0.04 to 0.10)	<0.001
Born outside UK/Ireland	0.21 (0.02 to 0.41)	0.029		
**Risk factors**				
Ever consumed alcohol	0.20 (0.01 to 0.40)	0.044		
Ever smoked	0.16 (−0.03 to 0.36)	0.105		
Ever used recreational drugs	0.42 (0.21 to 0.63)	<0.001		
**Body composition**				
Height in meters	1.68 (0.69 to 2.66)	0.001	−0.04 (−1.22 to 1.14)	0.944
**Blood pressure**				
Average MAP in mmHg	0.03 (0.02 to 0.05)	<0.001	0.03 (0.01 to 0.04)	<0.001
**Influencing factors on day of PWV**				
Caffeine 2 h before PWV	0.28 (0.01 to 0.56)	0.045		
Nicotine on day of PWV	0.57 (0.22 to 0.91)	0.001	0.52 (0.20 to 0.84)	0.001

** *All a priori variables, as well as those with univariable p < 0.15 and multivariable p < 0.05, are presented here*.

*PHIV, perinatal HIV; CI, confidence interval; MAP, mean arterial pressure; PWV, pulse wave velocity*.

**Table 4:** Univariable and multivariable predictors of PWV in PHIV group.

	**Univariable predictors**		Multivariable predictors^**^	
	**Coefficient (95% CI)**	***P-*value**	**Coefficient (95% CI)**	***P-*value**
Constant			3.16 (0.68 to 5.64)	…
**Sociodemographics**				
Male (vs. female)	0.41 (0.19 to 0.64)	<0.001	0.37 (0.10 to 0.65)	0.008
Age, per 1 year increase	0.08 (0.04 to 0.13)	<0.001	0.07 (0.03 to 0.11)	0.001
Born outside UK/Ireland	0.22 (−0.01 to 0.45)	0.062		
**Risk factors**				
Ever consumed alcohol	0.17 (−0.06 to 0.40)	0.147		
Ever used recreational drugs	0.36 (0.11 to 0.62)	0.006		
**Body composition**				
Height in meters	1.27 (0.11 to 2.43)	0.032	−0.54 (−1.94 to 0.86)	0.446
**Blood pressure**				
Average MAP in mmHg	0.03 (0.02 to 0.05)	<0.001	0.03 (0.01 to 0.04)	<0.001
**Influencing factors on day of PWV**				
Caffeine 2 h before PWV	0.39 (0.04 to 0.73)	0.030		
Nicotine on day of PWV	0.52 (0.11 to 0.93)	0.014	0.52 (0.13 to 0.91)	0.009
**HIV infection parameters**				
Time (years) on ART	0.01 (−0.01 to 0.03)	0.060		

** *All a priori variables, as well as those with univariable p < 0.15 and multivariable p < 0.05, are presented here*.

*PHIV, perinatal HIV; CI, confidence interval; MAP, mean arterial pressure; PWV, pulse wave velocity; ART, antiretroviral therapy*.

Table 4 presents univariable and multivariable predictors of higher (worse) PWV for the PHIV group only. Both before and after adjustment for other factors being male (ap = 0.008), older age (ap = 0.001), higher MAP (ap < 0.001) and nicotine use on the day of the PWV measurement (ap = 0.009) were associated with higher PWV. Being born outside of the UK/Ireland, ever consumed alcohol, ever used recreational drugs, height, consumed caffeine in the 2 h before the PWV measurements and longer duration of ART were all associated with worse PWV in univariable analyses but not in multivariable analyses, in the PHIV group. None of the other HIV-related variables considered, including CD4 cell count and the number of ART drugs/classes being taken at the time of the interview, and ever taken a protease inhibitor/abacavir/integrase inhibitor, were associated with PWV.

## Discussion

Our study assessed arterial stiffness *via* PWV in the largest study of young people living with and without PHIV to date measuring arterial stiffness. Compared to the HIV negative group we found worse PWV values in the PHIV group (ap = 0.020).

Other studies have had varying findings, and it is important to consider the different characteristics of the populations studied. Charakida et al. studied PWV in 83 children living with PHIV (48 on ART, mean age 11 years) and 59 HIV negative children (mean age 12 years) from England in 2009. They found significantly higher mean PWV values in the PHIV group than the HIV negative group, measuring carotid to radial PWV using the tonometric SphygmoCor system (5). The participants were recruited from one clinic in London, UK and therefore may also have participated in our AALPHI cohort.

Eckard et al. studied PWV in 101 young people living with HIV and on ART (median age 20 years) and 86 HIV negative young people (median age 19 years) from the USA between 2012 and 2014 (19). In contrast to Charakida et al., they found no significant difference in median PWV values between the two groups, measuring carotid to femoral PWV using the SphygmoCor system. Kuilder et al. studied PWV in 51 children living with PHIV and on ART (mean age 8 years) and 52 HIV negative children (mean age 9 years) from Indonesia in 2013. They also found no significant difference in mean PWV values between the two groups, measuring aortic PWV using the oscillometric Arteriograph system (20). Similarly, Kenny et al. studied PWV in 74 children living with PHIV and on ART (mean age 7 years) and 75 HIV negative children (mean age 7 years) from Africa in 2015. They found no significant difference in mean PWV values between the two groups, measuring carotid to femoral PWV using the Vicorder software (21).

There may be several possible reasons for the differing results of these studies. Firstly, different measurement devices, as well as measurement locations and methods of defining the distance that the pulse wave travels were used in the studies. There is no defined gold standard, especially for PWV measurements, which subsequently could lead to differing values (22). Secondly, the ages of the participants in the studies vary; Kuilder et al. and Kenny et al. included children who were considerably younger than the participants in our study for example (20, 21). While Charakida et al.'s study also included children, differences might be attributable to the differing physical measurement location (peripheral vs. central) (5). In adults, it has been proposed that different artery segments are susceptible to different risk factors, and that peripheral arteries show vascular changes earlier than the large, elastic central blood vessels and that functional changes occur in peripheral arteries first (6). Finally, in Eckard et al.'s study only half of the young people of had PHIV, so the median time living with HIV was only 8 years and likewise the median duration on ART was 3 years (19).

Previous studies have found increased (worse) intima-media thickness (IMT) and decreased (worse) flow-mediated dilation (FMD) in PHIV young people compared with HIV negative young people (23, 24). PWV is a marker of arterial stiffness (a functional marker of the vasculature) whereas IMT (usually measured at the carotid artery) is a marker of anatomic changes of the vascular wall and FMD is a marker of endothelial dysfunction, a precursor of atherosclerosis. Therefore, the results of our study may be an indication that the anatomic changes as seen with the increased IMT have led to functional changes of the vasculature and arterial stiffness in young people.

In our analyses male sex, older age and higher MAP were predictors of worse PWV and have previously been found to be important determinants of PWV in adults as well as children (5, 19, 22, 25). Nicotine use on the day of the PWV measurements was also associated with higher PWV in our study. Recent nicotine use, as well as chronic smoking, have been associated with increased arterial stiffness in adults (26). Chronic smoking might not have been significantly associated with PWV in our study due to reporting as “ever having smoked” rather than calculation of pack years or the cumulative effect of smoking duration.

While ART causes viral suppression and a reconstitution of the immune system and could therefore be seen as cardioprotective, previous studies have found a negative association between lipid and glucose levels and some ART drug classes such as protease inhibitors (27). In our analyses the duration on ART was a predictor for worse PWV measurements in univariable analyses but not in multivariable analyses, and ever having taken a protease inhibitor was not associated with PWV in the PHIV group. Approximately two-thirds of the total cholesterol and triglyceride values (only available for the PHIV group) were at acceptable levels in our study, with no association with PWV. Van den Hof et al. found no difference in total cholesterol or triglyceride levels in 35 PHIV (median age 13.8 years) taking ART and 37 HIV negative young people (median age 12.1 years). However, 60% of PHIV had elevated lipoprotein(a) levels which were significantly higher than the HIV negative group (28). Other studies have also shown signs of lipodystrophy as well as increased lipid levels in children and adolescents that have previously already been seen in adults (23, 27).

Our study has a number of limitations. Firstly, we were unable to compare our data to appropriate age/height/sex adjusted reference values. Few normative data are available for PWV in children and adolescents, and those that are available are based on specific measurement devices and more importantly on the method of defining the distance that the pulse wave travels (22). Secondly, although the number of participants in our study was greater than in previous published studies, the sample size was still relatively small and, in particular, our HIV negative group is comparably smaller than the PHIV group, which may affect the precision of estimates in our models. Thirdly, some of the data reported (adherence, previous medical conditions, family history of CVD or metabolic changes) were self-reported, and finally not all data were available for all participants (e.g., lipid measurements) as they were extracted from the national surveillance study CHIPS.

As the etiology of CVD in those living with HIV is multifactorial, studying PHIV could provide another piece in the puzzle of assessing the relative impact of HIV itself, ART and traditional cardiovascular risk factors in the development of CVD in people living with HIV. One of the advantages of studying people living with perinatal HIV is the known duration of HIV infection in comparison to people living with acquired HIV.

Longitudinal studies are becoming increasingly important to understand how the risk of chronic conditions change as PHIV age and change their behavior, and in particular as they transition into adult care. As recommended in current guidelines, a detailed history of cardiovascular risk factors, regular blood pressure and anthropometric measurements, as well as blood glucose levels and lipid panels should become a standard of care (8, 29). However, the optimal management of traditional risk factors remains unclear, with variation in guidelines for thresholds for pharmacological intervention such a lipid lowering agents and antihypertensives in the general youth population (17). For those living with HIV, careful consideration of the benefits of adjunctive therapies needs to be balanced against the risks of higher pill burden and potential detrimental effects on ART adherence, which could result in viral rebound and indeed subsequent cardiovascular risk. Similarly in terms of current ART regimens, there is a balance between adverse lipid profiles associated with boosted protease inhibitors, and potential weight gain with second generation integrase inhibitors (30). As it remains uncertain if and how cardiovascular risk factors will translate into actual outcomes of CVD, and if this will change with novel treatment regimens, cohort studies are required as the first generation living with lifelong HIV enter their fourth decade of life.

## Data Availability Statement

The datasets presented in this article are not readily available because the AALPHI data are held at MRCCTU at UCL, which encourages optimal use of data by employing a controlled access approach to data sharing, incorporating a transparent and robust system to review requests and provide secure data access consistent with the relevant ethics committee approvals. All requests for data are considered and can be initiated by contacting mrcctu.datareleaserequest@ucl.ac.uk. Requests to access the datasets should be directed to mrcctu.datareleaserequest@ucl.ac.uk.

## Ethics Statement

This study, involving human participants, was reviewed and approved by the Leicester Research Ethics Committee. Participants provided written informed consent, except where they lacked the capacity, in which case parents or guardians provided written informed consent and the young person provided written assent. Young people younger than 18 years were allowed to consent to participation in the study themselves if they were deemed by the study research nurses as having the capacity to consent.

## Author Contributions

MLP and KS acquired and verified the data. JM and HC did the analysis. JM and HC drafted the article, with major contributions from AJ and CF. All authors substantially contributed to the concept and design of the study, revised the article for important intellectual content, approved the final version for publication, and accountable for all aspects of the work.

## Funding

This work was supported by the Monument Trust and Penta Foundation. The MRC Clinical Trials Unit at UCL is supported by the Medical Research Council Programme Grant MC_UU_12023/26.

## Conflict of Interest

AJ reports grants from Abbvie, Bristol Myers Squibb, Gilead Sciences, Janssen Pharmaceuticals and ViiV Healthcare through the PENTA Foundation, and from the European Commission, European and Developing Countries Clinical Trials Partnership, Gilead Sciences, International AIDS Society, NHS England, Medical Research Council and PENTA Foundation outside the submitted work. All monies were paid to her institution. The remaining authors declare that the research was conducted in the absence of any commercial or financial relationships that could be construed as a potential conflict of interest.

## Publisher's Note

All claims expressed in this article are solely those of the authors and do not necessarily represent those of their affiliated organizations, or those of the publisher, the editors and the reviewers. Any product that may be evaluated in this article, or claim that may be made by its manufacturer, is not guaranteed or endorsed by the publisher.
